# Integrating Digital Health into School Nursing for Food Allergy Management: A Systematic Review

**DOI:** 10.3390/children13010159

**Published:** 2026-01-22

**Authors:** Rita Nocerino, Flavia Lotito, Emma Montella, Roberto Berni Canani

**Affiliations:** 1 Departmental Area of Health Services, Unit of Health Services Organization, University Hospital Federico II, 80131 Naples, Italy; emma.montella@unina.it; 2 NutriTechLab, University of Naples “Federico II”, 80131 Naples, Italy; berni@unina.it; 3 Department of Biomedicine and Prevention, University of Rome “Tor Vergata”, 00133 Rome, Italy; 4 Independent Researcher, 65121 Pescara, Italy; flavialot3@gmail.com; 5 Department of Translational Medical Science, University of Naples “Federico II”, 80131 Naples, Italy; 6 European Laboratory for the Investigation of Food-Induced Diseases, University of Naples “Federico II”, 80131 Naples, Italy; 7 Task Force for Microbiome Studies, University of Naples “Federico II”, 80131 Naples, Italy

**Keywords:** food allergy, anaphylaxis, digital health, school nurse, e-learning, pediatric nursing, health literacy, public health education

## Abstract

**Highlights:**

**What are the main findings?**

- Digital health interventions in school settings consistently improve knowledge, preparedness, and self-efficacy in food allergy and anaphylaxis management among school staff, parents, and children.
- Evidence on direct clinical outcomes remains limited, with most studies focusing on educational and psychosocial effects rather than real-world emergency performance.

**What are the implication of the main finding?**

- Digital health can act as a structural enabler to reduce inequalities in school-based food allergy management, particularly in contexts without institutionalized school nursing services.
- Integrating digital tools into school health frameworks may strengthen preparedness, inclusion, and coordination between schools, families, and healthcare systems.

**Abstract:**

**Background:** Food allergy [FA] is a growing public health concern among school-age children, with schools and childcare/daycare settings representing high-risk environments for accidental exposure and anaphylaxis. **Objective:** To systematically review evidence on digital health interventions supporting FA education, prevention, and management in school settings. **Methods:** A systematic search of PubMed, Scopus, Web of Science, and CINAHL was conducted to identify studies published between January 2015 and December 2025 [PROSPERO CRD420251185553]. Eligible studies evaluated e-learning, mHealth, or web-based programs targeting school staff, parents, or students. **Results:** Sixteen studies met inclusion criteria. Digital health emerged as a catalyst for professional development, interprofessional communication, and health equity within school communities. Interventions consistently improved knowledge, preparedness, and self-efficacy in anaphylaxis management among school staff, strengthened parental empowerment and communication with schools, and supported coping and inclusion among allergic children. Evidence on clinical outcomes; however, remains limited. **Conclusions:** Digital health can meaningfully enhance school preparedness and reduce inequalities in allergy management. Integrating digital tools into national school health frameworks—particularly where school nursing is not yet institutionalized—may represent a pivotal step toward safer, more equitable inclusion of children with food allergy.

## 1. Introduction

Food allergy [FA] is a major global public health challenge, with prevalence estimates ranging from 6% to 10% among school-aged children, and it remains one of the leading causes of anaphylaxis in this age group [1,2].

According to the World Health Organization and the International Council of Nurses, digital health encompasses not only e-learning and telemedicine but also data-driven and communication technologies supporting care coordination and health promotion [3,4]. Within nursing, digital health is recognized as a core competency that enhances evidence-based practice, patient empowerment, and interprofessional collaboration [4]. In educational contexts, this digital transition redefines the nurse’s role from a task-oriented caregiver to a digitally empowered educator and coordinator of health promotion. Digital health offers new opportunities to improve the identification, prevention, and management of FA and anaphylaxis in real-world environments such as schools [5]. These opportunities are particularly relevant in school settings, where digital tools can support nurses, teachers, and families by facilitating coordinated communication, timely decision-making, and standardized emergency preparedness.

The school and childcare/daycare setting represents a high-risk environment where accidental exposure to allergens is likely, and where preparedness, early recognition, and proper response are crucial for preventing severe allergic events [6,7]. International data indicate that approximately 10% of pediatric anaphylaxis occurs in schools, making them a priority target for structured prevention strategies [8].

School nurses play a key role in coordinating allergy care plans, supporting symptom self-management, providing education, and connecting schools with families and healthcare providers, especially in systems where their role is formally recognized [6,9]. However, in countries such as Italy, where the role of the school nurse is not yet institutionalized at national level, deep heterogeneity and fragmented practice models persist, generating inequalities in safety, access to training, and emergency preparedness across schools [10]. The focus of this review on school nurses reflects their distinctive position within school health systems. Unlike other health professionals involved in food allergy care—such as pediatricians, allergists, or dietitians—school nurses operate directly within the educational environment and are responsible for the day-to-day implementation of Individual Healthcare Plans, emergency preparedness, staff training, and communication with families. Their continuous presence at school and their dual educational and clinical role make them central actors in the effective translation of digital health interventions into real-world school practice. For this reason, the involvement of school nurses was prioritized in the present review.

Multiple studies have reported that school personnel frequently lack adequate and updated training in recognizing anaphylaxis, activating emergency protocols, and administering adrenaline auto-injectors, contributing to delayed response and increased risk [11]. Qualitative research has also shown that children and parents perceive schools as insufficiently prepared, with negative consequences on psychological well-being, sense of safety, and daily inclusion [12].

Digital health solutions—including e-learning for teachers, mobile apps for families, and web-based school protocols—are emerging as promising tools to standardize training, promote confidence in first-line management, and reduce geographical and organizational disparities [13]. Evidence demonstrates that digital programs can significantly improve knowledge, preparedness, and self-efficacy in managing FA and anaphylaxis, and may reduce anxiety and decisional uncertainty in newly diagnosed families [14].

Integrating digital health into school nursing practice therefore represents a strategic opportunity to support safe inclusion of allergic children, enhance quality of care, and harmonize clinical responses in educational environments—particularly in contexts where standard nursing models for school health are not yet uniformly implemented [15].

Despite the growing use of e-learning and mobile technologies for allergy education, no previous synthesis has specifically addressed how digital health interventions support school preparedness, parental empowerment, and psychosocial well-being of children with FA. Addressing this gap is essential because most educational systems worldwide, especially those lacking institutionalized school nursing services, rely heavily on digital solutions to compensate for fragmented allergy management structures [16].

This review aims to systematically synthesize current evidence on digital health interventions for food allergy management in school settings, highlighting implications for nursing practice and educational policy.

## 2. Materials and Methods

This systematic review was conducted according to the Preferred Reporting Items for Systematic Reviews and Meta-Analyses [PRISMA] 2020 guidelines [17]. Prior to study initiation, the research question, eligibility criteria, and analytic strategy were defined in advance in accordance with standards for evidence synthesis in health services research [18,19].

### 2.1. Protocol Registration

The protocol was prospectively registered in PROSPERO [registration number CRD420251185553].

### 2.2. Eligibility Criteria

Studies were eligible if they examined digital health interventions aimed at the prevention, recognition, or management of FA or anaphylaxis in school-aged children [0–18 years], their families, or school personnel. Studies were included if conducted in school or educational settings, or if they evaluated digital health interventions with direct implications for school preparedness, safety, or psychosocial adaptation of children with FA.

Digital health was operationalized according to the WHO definition and included online programs, e-learning modules, mobile health [mHealth], and web-based platforms [5]. Interventions relying solely on printed materials [e.g., PDFs or leaflets without interactive digital components] were not considered digital health. Eligible designs included randomized controlled trials, quasi-experimental studies, observational studies, qualitative and mixed-methods research, and systematic reviews. We excluded studies not conducted in school or educational contexts, not involving digital modalities, articles not available in English or Italian, and commentaries or abstracts without primary data [19]. Gray literature [unpublished reports, theses, conference abstracts without primary data] was not included in the review.

### 2.3. Information Sources and Search Strategy

The full search strategy for PubMed was as follows:

[“food allergy” OR “food hypersensitivity” OR “anaphylaxis”] AND [“school” OR “school nurse” OR “teacher” OR “educator”] AND [“digital health” OR “e-learning” OR “web-based” OR “online training” OR “mobile app” OR “mHealth” OR “information and communication technology”].

A comprehensive literature search was conducted in PubMed/MEDLINE, Scopus, Web of Science, and CINAHL to identify studies published between January 2015 and December 2025. 

A comprehensive literature search was conducted in PubMed/MEDLINE, Scopus, Web of Science, and CINAHL Boolean combinations of MeSH and keyword terms related to FA, anaphylaxis, school settings, and digital health were used. Search strategies were adapted to each database, and reference lists of included studies and relevant reviews were manually screened to identify additional eligible papers.

### 2.4. Study Selection

Two independent reviewers [R.N. and F.L.] screened titles and abstracts. All references were managed using Rayyan software (Rayyan Systems Inc., Cambridge, MA, USA) to facilitate blinded screening and automatic detection of duplicates. Full texts were retrieved for all potentially eligible studies and assessed against predefined inclusion and exclusion criteria. Disagreements were resolved through discussion or adjudication by a senior reviewer. Reasons for exclusion were recorded, and the selection process is reported in a PRISMA flowchart [17].

### 2.5. Data Extraction

Data were extracted independently by two reviewers using a standardized form. Extracted variables included country, population characteristics, study design, type of digital intervention, involvement of school nurses, and main outcomes. When necessary, authors were contacted for clarification of uncertainties or missing data.

### 2.6. Quality Appraisal

The methodological quality of the included studies was critically assessed by two independent reviewers [F.L. and R.N.] using the Joanna Briggs Institute [JBI] Critical Appraisal Tools [JBI, 2020] [20,21]. The specific JBI checklists were applied according to study design: randomized controlled trials [Checklist 1], quasi-experimental studies [Checklist 2], qualitative research [Checklist 3], cross-sectional surveys [Checklist 4], clinical guidelines [Checklist 5], systematic reviews [Checklist 6], and narrative [Checklist 7].

Each criterion within the checklists was rated as “Yes” [+] or “No” [−]. Studies meeting ≥80% of the criteria were classified as high quality, those meeting ≥50% as moderate quality, and those below 50% as low quality. Disagreements between reviewers were resolved through discussion until consensus was reached [22].

Overall, randomized controlled trials and cross-sectional surveys showed high methodological quality, particularly regarding clarity of inclusion criteria, validity of outcome measures, and appropriateness of statistical analyses. Quasi-experimental and qualitative studies demonstrated moderate to high rigor, though some lacked detailed reporting on follow-up procedures or reflexivity. Narrative and non-systematic reviews exhibited lower methodological robustness, mainly due to insufficient description of search strategies and appraisal procedures.

Among quasi-experimental studies, Allergy Pals [23] presented moderate methodological rigor, with clear outcome measures for self-efficacy but limited reporting on school-based implementation. Although not conducted in educational institutions, its focus on peer interaction and coping skills was considered relevant for the psychosocial dimensions of school inclusion.

The detailed results of the quality assessment for each study type are summarized in Table 1, Table 2, Table 3, Table 4, Table 5, Table 6 and Table 7.

### 2.7. Synthesis Approach

Given heterogeneity in populations, intervention components, and outcomes, a narrative synthesis was conducted. No meta-analysis was performed. Reporting followed PRISMA 2020 recommendations and methodological guidance from the Joanna Briggs Institute Manual for Evidence Synthesis [21].

## 3. Results

A total of 3627 records were identified through database searches [PubMed, Scopus, Web of Science, and CINAHL]. After the removal of 452 duplicates, 3175 records were screened based on titles and abstracts. Of these, 3123 were excluded because they did not meet the inclusion criteria. The full text of 52 reports was retrieved for detailed evaluation; 35 were excluded because the full text could not be accessed, 27 for wrong outcome, 6 for wrong population, and 2 for inappropriate study design. After full-text screening, 17 reports were assessed for eligibility, and 1 was excluded as not pertinent to the research question. Ultimately, 16 studies met the inclusion criteria and were included in the synthesis. Clinical guidelines and narrative reviews were included to contextualize empirical findings within existing policy and practice frameworks and were not considered primary sources of effectiveness evidence.

**Figure 1:**
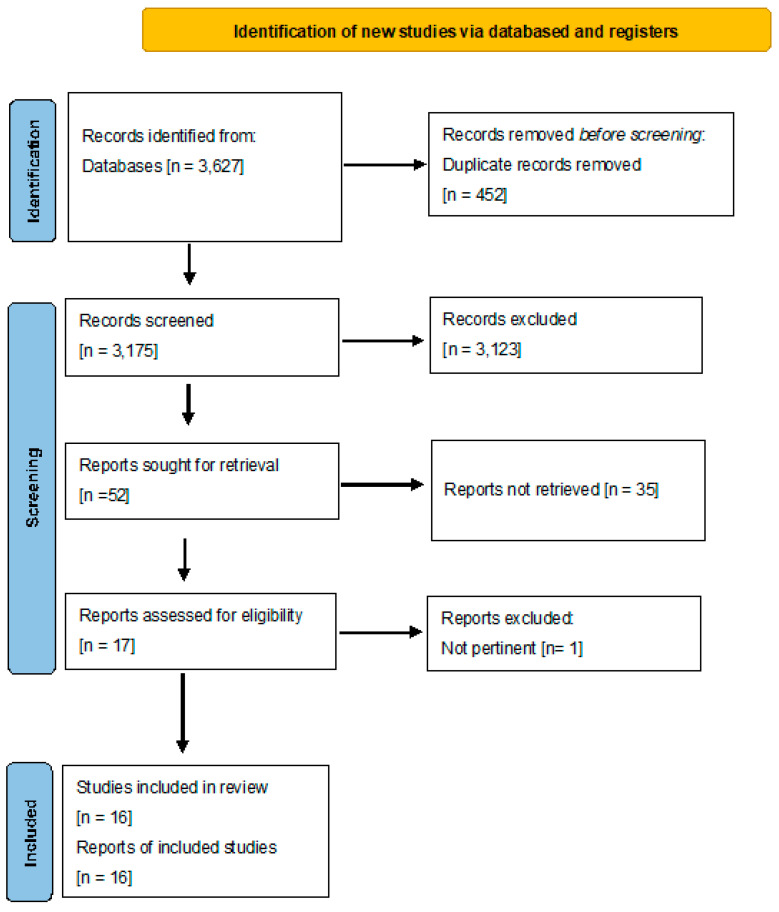
Flow diagram Prisma 2020.

The study selection process, including identification, screening, eligibility, and inclusion phases, is illustrated in Figure 1 [PRISMA 2020 flow diagram].

A total of 16 studies published between 2017 and 2025 were included: 3 randomized controlled trials, 3 quasi-experimental studies, 2 qualitative investigations, 5 cross-sectional surveys, 1 systematic review, 1 clinical guideline, and 1 narrative review. Sample sizes ranged from 16 to 170,000 participants. Across the included studies, three main thematic domains emerged: digital interventions improving school staff preparedness; digital tools supporting parental empowerment and family psychological outcomes; and child-facing and peer-based digital interventions promoting coping, inclusion, and equity in school environments [14,24].

A total of 16 studies met the inclusion criteria and were included in the synthesis. Most studies were conducted in North America, Europe, and East Asia and adopted quasi-experimental, cross-sectional, qualitative, or randomized controlled designs. Online educational interventions, mobile health platforms, and web-based training programs represented the predominant digital modalities described across studies [13,22].

The main characteristics of the included studies are summarized in Table 8, detailing country, population, study design, digital intervention type, and main outcomes.

### 3.1. Digital Health Interventions Improving School Staff Preparedness

Several studies demonstrated that digital programs significantly increased knowledge, perceived preparedness, and confidence in the recognition and management of anaphylaxis among school personnel [13]. Online education modules enhanced post-test performance and self-efficacy in correct epinephrine auto-injector use, with high completion and satisfaction rates [22]. Comparative evidence suggested that online training is effective in increasing theoretical knowledge, although simulation-based approaches may provide superior skill acquisition for operational emergency response competencies [11]. Overall, digital health solutions emerged as scalable modalities to standardize professional knowledge in heterogeneous educational environments where baseline preparedness varies considerably [28].

Collectively, these findings suggest that scalable, digitally mediated training can standardize allergy preparedness across schools, reduce variability in staff competence, and ultimately improve children’s safety at school [26].

### 3.2. Digital Tools Supporting Parental Empowerment and Family Psychological Outcomes

Digital interventions targeting parents improved health literacy, confidence, and management behaviors following diagnosis and during the adaptation phase of FA care. Web-based education for caregivers was associated with increased parental knowledge and high acceptability [29]. Studies based on interviews with parents and children show the need for more consistent school strategies, supported by digital tools, to improve the prevention and management of allergies [27]. Parental empowerment and advocacy behaviors for school safety were predicted by functional health literacy and by the strength of the parent–school communication relationship, highlighting the role of digital communication channels in facilitating shared safety processes [31,32]. Randomized digital health interventions designed to reduce psychological burden showed potential to mitigate anxiety, fatigue, and emotional distress in caregivers of newly diagnosed children, particularly when mobile tools integrated psycho-educational coping components [14].

### 3.3. Peer-Based and Child-Facing Digital Interventions Enhancing Inclusion and Coping

Child-facing digital interventions, including the *Allergy Pals* online peer-mentoring program [23], demonstrated significant improvements in children’s self-efficacy and emotional coping. Although the intervention was delivered in a community rather than a school setting, it specifically addressed school-related challenges such as peer interactions, stigma, and anxiety management. These findings indicate that peer-based digital approaches may complement school health strategies by reinforcing psychosocial resilience beyond the classroom. Qualitative research further confirmed that children perceive schools as environments with variable levels of safety, highlighting the need for digital resources that foster awareness, reduce misinformation, and promote social inclusion [12]. The outcomes related to inequalities show that digital tools can promote equity, as studies conducted in disadvantaged urban schools, that highlighting barriers such as a lack of school nurses and limited availability of epinephrine, indicate that digital technologies can expand training and provide standardized resources even in resource-poor settings. [28,29].

The study by Dupuis et al., conducted among adolescents, shows that combining digital messages with financial incentives doubles the likelihood of carrying an EAI [33]. The study on Individual Healthcare Plans for allergic children at school does not directly assess digital tools, but it highlights organizational shortcomings that could be significantly improved through digital solutions, such as digital platforms to standardize management plans, apps to facilitate the localization of emergency kits, digital tools for continuous staff training, and computerized systems to monitor allergic reactions [8].

Collectively, the evidence suggests that digital interventions targeting children can play a dual role in pediatric FA management: enhancing both self-regulation skills and social adaptation within educational contexts.

Collectively, the findings indicate that digital health interventions at school level primarily strengthen knowledge, preparedness, and empowerment across school staff, families, and affected children, while evidence for direct clinical outcome impact remains limited across the current literature base [29]. Importantly, most studies assessed outcomes immediately post-intervention or in the short term, with limited follow-up data on long-term retention, sustained behavior change, or emergency performance in real-life school settings.

## 4. Discussion

This systematic review shows that digital health interventions can significantly enhance preparedness, knowledge, and self-efficacy in the recognition and management of FA and anaphylaxis within school settings [14]. Digital training programs for school personnel demonstrated consistent effectiveness in improving theoretical competence regarding emergency protocols and administration of adrenaline auto-injectors, indicating that scalable online platforms may represent a sustainable solution to address heterogeneous baseline preparedness [23]. However, while online modules increase declarative knowledge, simulation-based learning appears superior for procedural skill acquisition and critical thinking in real-time emergency response [11]. Overall, the effectiveness of digital health interventions appears to be context-dependent. Asynchronous e-learning and web-based programs are particularly effective for improving theoretical knowledge, awareness, and confidence among large and heterogeneous groups, whereas simulation-enhanced or blended approaches are more suitable when procedural skills, critical thinking, and emergency response performance are required. Moreover, digital interventions alone may be insufficient to ensure sustained behavioral or organizational change if not embedded within supportive school policies, clear governance structures, and ongoing institutional commitment. These findings suggest that digital health is most effective when integrated into broader organizational and policy frameworks rather than implemented as isolated educational tools. Future research should move beyond single-arm knowledge outcomes and adopt comparative effectiveness designs to determine which educational modalities—including asynchronous e-learning, simulation-based training, blended formats, peer mentoring, and mobile coaching—are most effective for specific stakeholder groups. Teachers and school personnel may benefit from scalable standardized modules, whereas school nurses and designated responders may require simulation-enhanced training to consolidate procedural and decision-making skills. Similarly, parents, children, and adolescents may require tailored digital components addressing communication, coping, stigma reduction, and adherence behaviors. Stratified and implementation-oriented trials are needed to inform evidence-based allocation of training resources and to ensure both theoretical and operational competence in school settings.

Countries with institutionalized school nursing systems—such as Canada, the United Kingdom, and Finland—report higher preparedness levels and more consistent implementation of anaphylaxis protocols [14,27]. By contrast, Mediterranean countries, including Italy, face structural gaps in school-based health governance, making digital health an essential tool for bridging interregional disparities in allergy safety and education.

Digital health also has relevance in the psychosocial dimension of pediatric FA, particularly through parental empowerment and emotional support after diagnosis [24,29]. Interventions targeting parental health literacy and communication demonstrated improvement in advocacy, confidence, and shared decision-making with schools, revealing that digital health can facilitate relational safety-building processes between families and educational institutions [15]. Taken together, the findings illustrate a conceptual model in which digital health acts as a structural equalizer: it enhances preparedness through standardized education, strengthens communication pathways among schools, families and healthcare professionals, and promotes psychosocial well-being by reducing uncertainty and supporting children’s self-management. Randomized and quasi-experimental designs testing mobile health tools also suggest that digital resources can reduce anxiety, distress, and caregiver fatigue when FA management becomes an everyday burden [14]. These findings are aligned with broader literature documenting the substantial psychological load associated with pediatric FA and the need for scalable mental health support models [29]. From a nursing perspective, these results underscore the evolving educational and counseling role of nurses, who can act as mediators of digital knowledge transfer between families, schools, and healthcare services.

Child-facing digital interventions represent an emerging and underdeveloped area of research. Although online peer-mentoring experiences support coping and social inclusion, few studies have evaluated direct behavioral change or reduction in stigma in the school environment [26]. Qualitative research consistently demonstrates that children perceive schools as variable in preparedness and safety, suggesting the need for digital interventions that are not only educational but also social—designed to change peer norms and prevent exclusion or marginalization [12].

Notably, some digital interventions originally designed outside the school setting, such as the *Allergy Pals* peer-mentoring program [23], provide valuable insights into how virtual peer support can enhance coping, social inclusion, and readiness to manage allergies in educational contexts. This highlights the potential for integrating community-based and school-based digital tools within a continuum of care for children with FA.

Despite these promising findings, the current evidence base remains fragmented across countries, methodologies, and outcome domains—notably with limited data on real-world clinical outcomes such as reduced reaction rates or improved time-to-epinephrine administration [8]. Notably, most included studies primarily assessed educational and psychosocial outcomes, such as knowledge acquisition, perceived preparedness, confidence, and self-efficacy. In contrast, evidence on real-world clinical and implementation outcomes—including emergency response performance, time to epinephrine administration, or reduction in allergic reaction severity or frequency—remains scarce. This gap suggests that improvements in educational outcomes do not automatically translate into measurable clinical impact unless supported by structured implementation strategies. Future research should therefore prioritize implementation-focused designs capable of linking digital education to observable changes in school-based emergency management and health outcomes. An additional priority concerns the integration of structured feedback and monitoring systems into digital health interventions. Beyond knowledge transfer, digital programs should incorporate continuous quality-improvement loops, enabling schools and healthcare teams to monitor training uptake, protocol adherence, and real-world readiness. Examples of measurable indicators include periodic skills reassessment, adherence to Individual Healthcare Plans, audit of emergency kit availability, response pathway fidelity, time-to-epinephrine during drills or real events, and reporting of allergic incidents. Embedding dashboards, reminders, and standardized reporting within platforms may support sustained implementation and help translate educational gains into organizational and clinical impact. This is consistent with international observations that evidence for digital health effectiveness in allergy is presently more robust for knowledge-based dimensions than for measurable changes in emergency care performance [7].

From a policy and health systems perspective, this review highlights a major strategic opportunity for countries where the school nurse role is not fully institutionalized—such as Italy. The absence of a standardized national school nursing framework creates marked interregional inequities in preparedness, emergency competencies, and support to allergic students [10]. In this context, digital health can serve as a “knowledge equalizer,” enabling homogeneous access to training, standardized emergency protocols, shared educational materials, and interoperable communication pathways between families, teachers, and healthcare professionals—even in the absence of full-time nurses in each school [5]. This equalizing function reinforces the nurse’s responsibility not only as a clinical professional but also as a policy stakeholder, promoting equitable access to evidence-based allergy care. Integrating structured digital platforms within regional school health programs may represent a decisive step toward harmonizing emergency preparedness, standardizing Individual Healthcare Plans, and supporting interprofessional collaboration even in schools without on-site nurses. Although Italy is discussed as a paradigmatic case, comparable gaps in school health governance and allergy preparedness have been reported in other countries lacking standardized school nursing systems. The Italian context was therefore used as an illustrative example to contextualize the potential of digital health as a scalable and transferable solution.

The adoption of digital health tools in school settings must also align with ethical and deontological principles of nursing practice. According to the Italian Nursing Code of Ethics [30], digital competence and communication are integral to professional responsibility and to safeguarding informed consent, privacy, and equity in access to care. Ethical reflection must therefore accompany every stage of digital implementation, ensuring that innovation remains person-centered and consistent with nursing’s humanistic values.

The European Academy of Allergy and Clinical Immunology [EAACI] has emphasized that school preparedness for anaphylaxis must combine prevention, competence, and system-level harmonization, rather than isolated educational acts [34]. Digital health aligns with this principle because it supports not merely training but also standardization, remote updating, and system-wide reach, which are core to sustainable public health implementation [33]. This convergence underscores a paradigm shift: digital health is not an accessory to school nursing—it is a structural enabler of equitable FA safety.

Although this systematic review provides a comprehensive synthesis of current evidence, some limitations must be acknowledged. The studies included were heterogeneous in terms of design, population, intervention type, and outcomes assessed, which made it impossible to conduct a meta-analysis. Most investigations focused on short-term educational effects—such as increased knowledge and self-efficacy—without evaluating the long-term sustainability of behavioral or organizational changes. In addition, only a few studies reported objective clinical indicators, such as reduced incidence of allergic reactions or improved response times during anaphylaxis. This limited longitudinal assessment constrains the ability to determine which training approaches produce durable competencies and whether digital education translates into measurable improvements in emergency response and clinical outcomes over time. The possibility of publication bias toward studies with positive results cannot be entirely excluded, as well as language bias, given that only articles published in English and Italian were considered. An additional limitation is the relatively small number of studies included in the final synthesis, which reflects the still emerging nature of digital health interventions for FA management in school settings and may limit the generalizability of the findings. Furthermore, the limited representation of low- and middle-income countries restricts the global generalizability of the findings and highlights the need for context-specific adaptation of digital health strategies.

Nevertheless, this review also has important strengths. It represents, to our knowledge, the first systematic synthesis addressing digital health interventions for FA allergy management specifically within the school context, integrating educational, psychological, and policy perspectives through a nursing lens. By combining quantitative and qualitative evidence, it provides a multidimensional understanding of how digital health can support both clinical preparedness and psychosocial inclusion of children with FA. Moreover, the review highlights a relevant gap in the Italian school health system and proposes digital health as a strategic lever for promoting equity, interprofessional collaboration, and the modernization of nursing practice in educational environments.

## 5. Conclusions

This systematic review highlights that digital health represents a transformative opportunity to enhance preparedness, safety, and psychosocial well-being for children with FA in educational environments. Digital interventions—ranging from e-learning and web-based modules for teachers to mobile health platforms for families—have consistently shown improvements in knowledge, confidence, and self-efficacy across multiple stakeholders, including school staff, parents, and affected students [13,22,24]. These gains, while primarily educational, form the foundation for more resilient systems of allergy management, aligning with the World Health Organization’s call for digital transformation to strengthen public health infrastructure [5].

The evidence suggests that digital training can partially compensate for the absence of on-site healthcare professionals by equipping teachers and caregivers with essential competencies in prevention and first response [7]. Nevertheless, online education alone is insufficient to ensure real-world readiness unless integrated into structured organizational frameworks supported by healthcare authorities and educational institutions [9]. Effective preparedness therefore requires a combined approach that merges digital innovation with clear policy mandates, local leadership, and interprofessional collaboration between schools, nurses, and allergy specialists [35].

For Italy, where the role of the school nurse is still regionally fragmented and not yet institutionalized, digital health could act as an equalizer—standardizing competencies, disseminating protocols, and facilitating remote collaboration across educational and healthcare systems [10]. The integration of digital training and communication tools within regional school health programs would allow equitable access to evidence-based allergy management practices, supporting the European vision of inclusive and safe schools for all children [33].

Future research should focus on evaluating implementation outcomes, including sustainability, acceptability, and cost-effectiveness of digital interventions in diverse educational systems [34]. Mixed-methods approaches combining quantitative impact metrics with qualitative insight into user experience and contextual barriers are needed to guide large-scale adoption. Particular attention should be given to designing interventions that extend beyond knowledge transfer to address behavioral and organizational change, ultimately embedding digital health as a structural component of school-based chronic disease management [32].

In conclusion, digital health has the potential to bridge existing gaps in allergy preparedness, promote equity across educational systems, and strengthen the continuum of care between health and education sectors. For healthcare professionals—and particularly for nurses, who represent the primary health professionals operating within schools—this evolution offers a unique opportunity to redefine their role as digital facilitators of safety, inclusion, and empowerment in the everyday lives of allergic children.

Ultimately, integrating digital health into school nursing practice represents not only a technological advancement but a paradigm shift toward safer, more inclusive, and equity-driven educational environments for all children. Future large-scale implementation should be guided by structured frameworks ensuring governance, sustainability, and measurable indicators of quality and preparedness.

Determining which education and training strategies are most effective for different caregivers and patient groups—and establishing measurable monitoring indicators—should be considered a priority for future research and large-scale implementation.

## Figures and Tables

**Table 1:** JBI Checklist for RCT Studies.

	Kwen H. et al. 2022—75% [24]	Dupuis R. et al. 2023—85% [25]	Broome B. et al. 2021—65% [14]
1. Was true randomization used for assignment of participants to treatment groups?	+	+	+
2. Was allocation to treatment groups concealed?	+	+	+
3. Were treatment groups similar at the baseline?	+	+	+
4. Were participants blind to treatment assignment?	−	−	−
5. Were those delivering the treatment blind to treatment assignment?	−	−	−
6. Were treatment groups treated identically other than the intervention of interest?	+	+	−
7. Were outcome assessors blind to treatment assignment?	−	+	+
8. Were outcomes measured in the same way for treatment groups?	+	+	+
9. Were outcomes measured in a reliable way	+	+	+
10. Was follow up complete and if not, were differences between groups in terms of their follow up adequately described and analyzed?	+	−	−
11. Were participants analyzed in the groups to which they were randomized?	+	+	+
12. Was appropriate statistical analysis used?	+	+	+

Each JBI checklist item was rated as ‘Yes’ (+) if the criterion was met and ‘No’ (−) if it was not met. The overall methodological quality of each study was calculated as the proportion of criteria satisfied.

**Table 2:** JBI Checklist for Quasi-Experimental Studies.

	Dhanjal R. et al. 2023—80% [26]	Kim Y. et al. 2025—90% [11]	Sharma B. et al. 2025—80% [13]
1. It is clear in the study what is the “cause” and what is the “effect” [i.e., there is no confusion about which variable comes first]?	+	+	+
2. Was there a control group?	−	+	−
3. Were participants included in any comparisons similar?	+	+	+
4. Were the participants included in any comparisons receiving similar treatment/care, other than the exposure or intervention of interest?	+	+	+
5. Were there multiple measurements of the outcome, both pre and post the intervention/exposure?	+	+	+
6. Were the outcomes of participants included in any comparisons measured in the same way?	+	+	+
7. Were outcomes measured in a reliable way?	+	+	+
8. Was follow-up complete and if not, were differences between groups in terms of their follow-up adequately described and analyzed?	−	−	−
9. Was appropriate statistical analysis used?	+	+	+

Each JBI checklist item was rated as ‘Yes’ (+) if the criterion was met and ‘No’ (−) if it was not met. The overall methodological quality of each study was calculated as the proportion of criteria satisfied.

**Table 3:** JBI Checklist for Qualitative Research Studies.

	Vollmer R. et al. 2022—100% [27]	Feldman L. et al. 2022—100% [12]
1. Is there congruity between the stated philosophical perspective and the research methodology?	+	+
2. Is there congruity between the research methodology and the research question or objectives?	+	+
3. Is there congruity between the research methodology and the methods used to collect data?	+	+
4. Is there congruity between the research methodology and the representation and analysis of data?	+	+
5. Is there congruity between the research methodology and the interpretation of results?	+	+
6. Is there a statement locating the researcher culturally or theoretically?	+	+
7. Is the influence of the researcher on the research, and vice- versa, addressed?	+	+
8. Are participants, and their voices, adequately represented?	+	+
9. Is the research ethical according to current criteria or, for recent studies, and is there evidence of ethical approval by an appropriate body?	+	+
10. Do the conclusions drawn in the research report flow from the analysis, or interpretation, of the data?	+	+

Each JBI checklist item was rated as ‘Yes’ (+) if the criterion was met and ‘No’ (−) if it was not met. The overall methodological quality of each study was calculated as the proportion of criteria satisfied.

**Table 4:** JBI Checklist for Cross-Sectional Survey Studies.

	Poza-Guedes P. et al. 2021—75% [22]	Koo L. et al. 2023—100% [15]	Ruiz-Baqués A. et al. 2018—75% [28]	Hogue S. et al. 2018—90% [23]	Pouessel G. et al. 2017—90% [8]
1. Were the criteria for inclusion in the sample clearly defined?	+	+	+	+	+
2. Were the study subjects and the setting described in detail	+	+	+	+	+
3. Was the exposure measured in a valid and reliable way	+	+	+	+	+
4. Were objective, standard criteria used for measurement of the condition?	+	+	+	+	+
5. Were confounding factors identified?	−	+	−	+	+
6. Were strategies to deal with confounding factors stated?	−	+	−	−	−
7. Were the outcomes measured in a valid and reliable way?	+	+	+	+	+
8. Was appropriate statistical analysis used?	+	+	+	+	+

Each JBI checklist item was rated as ‘Yes’ (+) if the criterion was met and ‘No’ (−) if it was not met. The overall methodological quality of each study was calculated as the proportion of criteria satisfied.

**Table 5:** JBI Checklist for Textual Evidence Policy Clinical Guidelines.

	Waserman S. et al. 2021 100% [7]
1. Are the developers of the policy/consensus guideline [and any allegiences/affiliations] clearly identified?	+
2. Do the developers of the policy/consensus guideline have standing in the field of expertise?	+
3. Are appropriate stakeholders involved in developing the policy/guideline and do the conclusions drawn represent the views of their intended users?	+
4. Are biases due to competing interests acknowledged and responded to?	+
5. Are the processes of gathering and summarizing the evidence described?	+
6. Is any incongruence with the extant literature/evidence logically defended?	+
7. Are the methods used to develop recommendations described?	+

Each JBI checklist item was rated as ‘Yes’ (+) if the criterion was met and ‘No’ (−) if it was not met. The overall methodological quality of each study was calculated as the proportion of criteria satisfied.

**Table 6:** JBI Checklist for Systematic Review.

	Knibb R. et al. 2024 100% [29]
1. Is the review question clearly and explicitly stated?	+
2. Were the inclusion criteria appropriate for the review question?	+
3. Was the search strategy appropriate?	+
4. Were the sources and resources used to search for studies adequate?	+
5. Were the criteria for appraising studies appropriate?	+
6. Was critical appraisal conducted by two or more reviewers independently?	+
7. Were there methods to minimize errors in data extraction?	+
8. Were the methods used to combine studies appropriate?	+
9. Was the likelihood of publication bias assessed?	+
10. Were recommendations for policy and/or practice supported by the reported data?	+
11. Were the specific directives for new research appropriate?	+

Each JBI checklist item was rated as ‘Yes’ (+) if the criterion was met and ‘No’ (−) if it was not met. The overall methodological quality of each study was calculated as the proportion of criteria satisfied.

**Table 7:** Checklist for Narrative Review.

	Bartnikas L. et al. 2022 100% [30]
1. Is the generator of the narrative a credible or appropriate source?	+
2. Is the relationship between the text and its context explained? [where when, who with, how]	+
3. Does the narrative present the events using a logical sequence so the reader or listener can understand how it unfolds?	+
4. Do you, as reader or listener of the narrative, arrive at similar conclusions to those drawn by the narrator?	+
5. Do the conclusions flow from the narrative account?	+
6. Do you consider this account to be a narrative?	+

Each JBI checklist item was rated as ‘Yes’ (+) if the criterion was met and ‘No’ (−) if it was not met. The overall methodological quality of each study was calculated as the proportion of criteria satisfied.

**Table 8:** Characteristics of eligible studies.

Author	Title	Year	Country	Population	Study Design	Aim	Summary	Type of Digital Intervention	Role of Nurse	Outcomes
Dhanjal R. et al. [26]	An online, peer-mentored food allergy program [Allergy Pals]	2023	USA	Children aged 7–11 with food allergies and their parents	Quasi-experimental study	To evaluate the impact of an online peer-mentoring program	Improved confidence and self-efficacy among children and parents; knowledge remained stable	Online peer-mentoring platform “Allergy Pals”	Educational support	Increased confidence, improved coping; no significant improvement in technical skills
Vollmer R. et al. [27]	A Qualitative Investigation of Parent and Child Perceptions	2022	USA	Parents and children aged 8–18 with food allergies	Qualitative study [interviews]	To explore perceptions regarding school policies and safety	Parents and children report inconsistencies and limited implementation of policies	Recruitment and data collection through online posts and surveys on Facebook and Qualtrics	Perceived as important figures but not consistently present	Perceived insecurity, anxiety, need for digital support
Hogue S. et al. [23]	Barriers to the Administration of Epinephrine in Schools	2018	USA	U.S. schools [n = 12,275]	Cross-sectional observational study	To analyze barriers to epinephrine use in schools	Many schools consider epinephrine first-line therapy, but organizational and training barriers delay administration	Web-based survey	Central role but limited by insufficient staffing	Identified organizational and educational barriers
Kim Y. et al. [11]	Comparison of simulation-based and online training for school nurses	2025	South Korea	School Nurse	Quasi- experimental study	To compare simulation-based vs. online training effectiveness	Simulation more effective for critical thinking and practice; online training useful for theoretical knowledge	High-fidelity simulation [SBAT] vs. online module [OBAT]	Direct recipients of training	Higher self-efficacy and skills with SBAT; better theoretical knowledge with OBAT
Kwen H. et al. [24]	Development and Evaluation of a Mobile Web-based Food Allergy Program	2022	South Korea	Parents of allergic children [n = 73]	Randomized Controlled Trial	To evaluate a mobile/web educational program	Increased knowledge, self-efficacy, and management behaviors	Mobile/web program [2-week modules: multimedia lessons, quizzes, webinars, coaching, Q&A] vs. paper booklet	Involved in design and educational support	Significant improvement in knowledge and behaviors
Ruiz-Baqués A. et al. [28]	Evaluation of an Online Educational Program for Parents and Caregivers	2018	Spain	Parents/caregivers [n = 207]	Cross-sectional study	To evaluate a 2-week online educational program	Increased knowledge and high satisfaction [8.7/10]	E-learning with videos, forums, chat	Involved in multidisciplinary training	Improved knowledge and satisfaction
Broome B. et al. [14]	Food Allergy Symptom Self-Management with Technology [FASST]	2021	USA	Caregivers of newly diagnosed allergic children	Randomized Controlled Trial [protocol]	To develop an app to reduce anxiety and improve self-management	mHealth app for psychosocial support, under testing	Mobile app	Potential facilitators of clinical implementation	Reduced anxiety, depression, caregiver fatigue
Poza-Guedes P. et al. [22]	Implementing ICT Education on Food Allergy and Anaphylaxis in Schools	2021	Spain	Students, teachers, school nurses	Cross-sectional study	To evaluate an ICT-based program in schools	Increased awareness and faster emergency response	ICT educational program	Key participants and actors in management	Improved knowledge and response time
Sharma B. et al. [13]	Online food allergy and anaphylaxis education for school personnel [AllergyAware]	2025	Canada	School personnel [170,000 users]	Descriptive observational study	To evaluate a national e-learning course	High satisfaction; strong post-training scores	“AllergyAware” asynchronous e-learning course	Trainers and users	95% pass rate; increased confidence in epinephrine use
Koo L. et al. [15]	Parental Health Literacy, Empowerment, and Advocacy for Food Allergy Safety in Schools	2023	USA	Parents of allergic children [n = 313]	Cross-sectional survey	To investigate parental health literacy and advocacy	Effective advocacy linked to empowerment and good school–family relationships	Online Qualtrics survey	Collaborators in school–family communication	Functional literacy predicts effective advocacy
Feldman L. et al. [12]	Children’s Perspectives on Food Allergy in Schools	2024	Canada	16 children [5–13 years], with/without allergies	Qualitative study	To explore children’s perceptions and knowledge	Poor peer knowledge; need for accessible educational programs	Educational videos	Collaboration in projects	Identified knowledge gaps; need for peer education
Bartnikas L. et al. [30]	Food Allergies in Inner-City Schools: Addressing Disparities	2022	USA	Children in U.S. urban schools	Narrative review	To explore disparities and challenges	Racial and socioeconomic disparities; lack of full-time school nurses	Online surveys on Qualtrics	Limited by scarce resources	Identified inequality in access to care and training
Dupuis R. et al. [25]	Food Allergy Management for Adolescents Using Behavioral Incentives	2023	USA	Adolescents [15–19 years], n = 131	Randomized Controlled Trial	To evaluate SMS + incentives to increase epinephrine carriage	SMS alone ineffective; financial incentives effective	SMS reminders + financial incentives	Indirect role but involved in education	EAI carriage: 45% [intervention] vs. 23% [control]
Waserman S. et al. [7]	Prevention and management of allergic reactions to food in care centres and schools: Practice guidelines	2021	International [USA, Canada, Ireland, Italy, Japan, Australia, Mexico]	Schools and childcare centres	Practice guidelines [GRADE-based]	To provide evidence-based recommendations	Recommendations include action plans, training, stock epinephrine; discourage total food bans	Dissemination of protocols	Central implementers	Practical recommendations for school safety
Knibb R. et al. [29]	Psychological support needs for children with food allergy and families: Systematic Review	2024	International [USA, UK, Italy]	838 participants [children, parents]	Systematic review	To examine psychological support and effective interventions	Professional-guided CBT effective; self-guided online CBT ineffective	Online CBT self-help programs	Collaboration in referral and support	Reduced anxiety and improved QoL with guided CBT
Pouessel G. et al. [8]	Individual healthcare plan for allergic children at school	2017	France	Allergic children in Northern French schools [n = 1325 IHPs]	Prospective observational study	To evaluate practical implementation of IHPs	Analyzed emergency kits, meal management, allergy reactions across a school year	Organizational/clinical documentation tool	Support in IHP management and staff training	70% emergency kits contained epinephrine; variable medications; 60 reactions [2 requiring epinephrine]

## Data Availability

No new data were created or analyzed in this study. Data sharing is not applicable to this article.
